# Soldering of Electronics Components on 3D-Printed Conductive Substrates

**DOI:** 10.3390/ma14143850

**Published:** 2021-07-09

**Authors:** Bartłomiej Podsiadły, Andrzej Skalski, Marcin Słoma

**Affiliations:** Micro- and Nanotechnology Division, Institute of Metrology and Biomedical Engineering, Faculty of Mechatronics, Warsaw University of Technology, 8 sw. A. Boboli st., 02-525 Warsaw, Poland; a.skalski@mchtr.pw.edu.pl (A.S.); m.sloma@mchtr.pw.edu.pl (M.S.)

**Keywords:** structural electronics, conductive composites, 3D printing, additive manufacturing, solder joint properties

## Abstract

Rapid development of additive manufacturing and new composites materials with unique properties are promising tools for fabricating structural electronics. However, according to the typical maximum resolution of additive manufacturing methods, there is no possibility to fabricate all electrical components with these techniques. One way to produce complex structural electronic circuits is to merge 3D-printed elements with standard electronic components. Here, different soldering and surface preparation methods before soldering are tested to find the optimal method for soldering typical electronic components on conductive, 3D-printed, composite substrates. To determine the optimal soldering condition, the contact angles of solder joints fabricated in different conditions were measured. Additionally, the mechanical strength of the joints was measured using the shear force test. The research shows a possibility of fabricating strong, conductive solder joints on composites substrates prepared by additive manufacturing. The results show that mechanical cleaning and using additional flux on the composite substrates are necessary to obtain high-quality solder joints. The most repeatable joints with the highest shear strength values were obtained using reflow soldering together with low-temperature SnBiAg solder alloy. A fabricated demonstrator is a sample of the successful merging of 3D-printed structural electronics with standard electronic components.

## 1. Introduction

Additive manufacturing, also called three-dimensional printing (3D printing), is a new manufacturing trend, allowing the fabrication of physical objects directly from the digital design. It is rapidly and dynamically evolving, in some cases replacing or supplementing conventional manufacturing techniques. 3D printing has many advantages like freeform design, easy customization, personalization of elements, a significant automatization level, or a lower waste of materials. Furthermore, it is also widely used in prototyping to reduce the time and cost of manufacturing new parts or small-quantity productions [1]. Additive manufacturing has been widely applied in many industries like automotive, architecture, fashion, aerospace, biomechanical [2,3,4,5].

The term additive manufacturing has a broad meaning, and comprises many techniques that use different materials and equipment, including Stereolithography (SLA), PolyJet, Selective Laser Sintering (SLS), and Fused Deposition Modeling (FDM), with the last one being the most popular nowadays [6]. The FDM technique is the trendiest due to its low-cost equipment, process simplicity, accessibility of low-cost materials, and acceptable printing resolution down to 50 µm [7]. Thanks to this, we are able to fabricate functional elements that cannot be made with conventional methods, or we can develop personalized elements with specific shapes and colors on customer demand. To achieve even more complex manufacturing using 3D printing, new materials for this technique are continuously being developed.

A good example is a new trend in manufacturing called structural electronics. Nowadays, most electronic devices are still manufactured using the PCB design due to the challenges of integrating new additive technology [8,9]. Structural electronics is a term used to name structures that involve electronic circuits and components in the volume of protective structures, housing elements, etc. [10,11,12]. Structural electronics can function as electronic circuits and components and provide the optimal mechanical and protective properties of the entire device. Additionally, reducing the volume of a conventionally fabricated device can be done by fusing additive manufacturing and electronics [13]. Due to the rapid development of this branch of manufacturing, there is a need to develop new materials with unique properties that can be used in Additive Manufacturing. Materials with excellent electrical properties like low resistivity or high dielectric constant, excellent mechanical properties, unique magnetic or thermal properties, etc., have to be developed to increase the number of structural electronics applications [14,15,16,17,18].

This paper presents the application of elaborated copper-based conductive filaments with a different polymer matrix for 3D structural electronics fabrication. Such filaments can be directly used in unmodified, low-cost FDM printers to fabricate electrical circuits and other functional conductive elements. To manufacture a sophisticated electrical device, we need to merge additive manufacturing methods with traditional PCB technology. Based on the approaches used so far, the only way to fabricate conductive connections on composite substrates is to use conductive adhesives. However, such connections are characterized by lower shear strength compared to solder joints [19]. Additionally, and crucial in structural electronics applications, conductive adhesives have lower electrical conductivity than solder alloys. This is because conductive adhesives are composite materials containing a non-conductive matrix that provide good adhesive properties. Only the functional phase found in the adhesives can provide electrical conductivity. In the case of solder alloys, they conduct throughout their volume [20,21,22,23]. In this paper, soldering tests of fabricated composites were performed to obtain optimal process conditions for solder connections on polymer composites. Two different soldering methods and two types of solder alloys were used in the research: reflow and iron soldering were employed, along with SnPb and low-temperature SnBiAg solder alloys, respectively. The shear strength test of the fabricated solder connections was determined. This kind of research is being reported for the first time in the literature. Additionally, the 3D-printed demonstrator incorporating LED elements is presented.

## 2. Experimental Procedure

### 2.1. Materials

**Figure 1:**
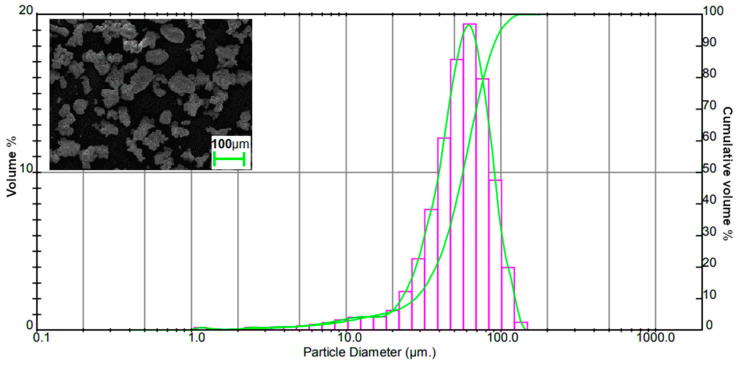
Particle size distribution and SEM micrograph of copper powder.

To fabricate a conductive composite filament for the FDM technique, two materials need to be incorporated: polymer matrix and conductive filler. The bulk copper’s conductivity is 5.96 × 10^7^ S/m, which is only slightly lower than silver—6.14 × 10^7^ S/m. Taking into consideration the significant weight of metal powder necessary to reach the electrical percolation threshold in the composites and the cost of such material, it was decided to choose copper micro powder as a filler. The metal powder was purchased from Makin Metal Powder (Rochdale, Lancashire, United Kingdom). According to the datasheet, the purity of copper powder was above 99%. To examine the average particle size of the filler, particle size analysis was performed on the Malvern machine. Grain size distribution and SEM picture of copper powder are shown in Figure 1. The average filler particle diameter was calculated as 57 μm.

To develop composite filament that can be used in FDM printing, three different polymers were chosen as the matrix material to achieve the best possible processability. All selected thermoplastic polymers have different properties, but all are used in 3D printing: acrylonitrile butadiene styrene (ABS), polylactic acid (PLA), and polystyrene (PS).

Two different soldering alloys were used to prepare soldering tests. Sn63Pb37 is a eutectic alloy. It has a melting temperature of 187 °C, and it solidifies rapidly at one temperature rather than over the range, which can take place for non-eutectic alloys. In the experiment, Sn63Pb37 alloy was used in the form of a 1-mm-diameter wire. The second soldering alloy used in tests was low-temperature bismuth-based soldering paste. The OM 520 paste consists of 42% tin, 57.6% bismuth, and 0.4% silver. OM 520 solder paste was used because of its low melting temperature—138 °C—allowing it to be used in a low temperature soldering process. It is essential for soldering on thermally sensitive substrates not resistant to high temperatures.

### 2.2. Conductive Composite Fabrication

Metal–polymer composite filaments can be fabricated using two approaches. The first way is the thermal mixing of polymers with metal powder. The polymer is plasticized at high temperature and mixed with metal powder to obtain a homogeneous composite. This method has disadvantages, because to achieve high material homogeneity, the polymer needs to be melted at a high temperature for a long time. This process causes the thermal degradation of the polymer, leading to the deterioration of the composite’s mechanical properties. The second method used in this research is a two-stage solvent assisted processing method. The first stage of this method is dissolving the polymer in a suitable solvent. After that, the metal filler is added and mixed with the polymer. The last step is the evaporating of the solvent. This method allows minimizing the thermal degradation of the polymer during the fabrication of the composite. After solvent evaporation, the obtained composite is pelletized. A single-screw extruder machine was used to fabricate filaments from prepared composite pellets. Composites were extruded in a hot mixing extrusion process. Most popular 3D printers use filaments with 1.75 mm diameter. Our laboratory has extruded filaments with diameter values ranging from 1.7 mm to 1.8 mm, which is acceptable for use in standard, non-modified FDM printers. A detailed description of the fabrication of copper-based conductive filaments and the evaluation of electrical properties with the percolation threshold calculation has been presented in our previous work [24].

For the soldering tests, composites with the optimal amount of copper powder, ABS/Cu with 81.8 wt.%, PLA/Cu with 82.5 wt.% and PS/Cu with 85.5 wt.% of copper powder were used, respectively. All composite elements were printed on the CTC Bizer 2X 3D printer. Layer height was set to 0.3 mm, table temperature to 80 °C, and printing speed to 40 mm/s. The nozzle temperature value was different for each composite, depending on polymer matrix—for ABS/Cu composite, printing nozzle temperature was set to 250 °C, for PLA/Cu composite to 160 °C, and for PS/Cu composite to 150 °C.

### 2.3. Soldering Methods

Two methods of soldering were tested—hot iron soldering and reflow soldering. Hot iron soldering is a soldering method characterized by low repeatability but is widely used in prototyping, small-volume production, and service repair. It is the most common and easiest method of soldering. To fabricate the solder joint, soldering iron generates the heat required to increase the temperature of the soldered elements and circuit pads, and melt the solder. On the other hand, reflow soldering is a common technique used in mass production and can be used to fabricate a large number of soldered connections at once. To produce solder connections with this method, surface-mount components and soldering paste are placed on the substrate and inserted into the reflow oven. The most important feature of this method is that the substrate and solder paste slowly heats up to a temperature slightly above the solder alloy’s melting point.

### 2.4. Composite Surface Preparation Methods

The research results described in this article are divided into four parts. This is due to the use of two types of soldering alloys (SnPb and SnBiAg) and two methods of fabrication of soldered joints (hand soldering and reflow soldering). Regardless of the chosen solder and soldering method, the research was carried out on three types of composite substrates-ABC/Cu, PLA/Cu, and PS/Cu. Composite substrates were prepared for the soldering process in five different ways, according to Table 1. This allows us to determine the impact of the composite surface preparation methods on the quality of soldered joints.

## 3. Results and Discussion

### 3.1. Solder Wettability Test

Wettability is the ability of a material, typically a liquid, to spread over another material. It is crucial for soldering because it plays an essential role in providing a good connection between the solder element and the substrate. One of the solder wettability measurements on different substrates is measuring the contact angle (θ). It has been proven that the smaller the value of the contact angle, the better the wettability [25]. Good wettability has a direct impact on solder joint properties, like mechanical strength and electrical resistance. The contact angle depends on many factors, like the soldering method, soldering alloy, substrate surface preparation, etc.

**Figure 2:**
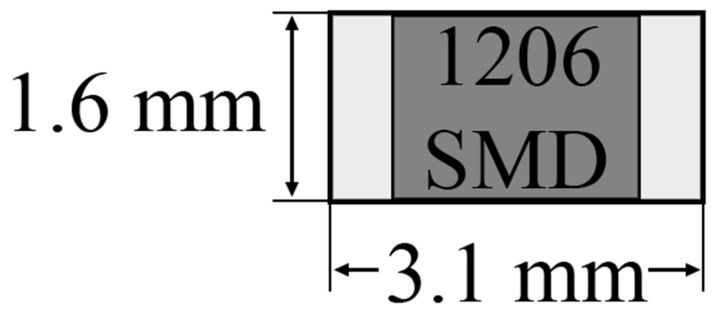
The schematic of SMD resistor with dimensions.

In the first part of this research, the SnPb solder alloy and hot iron soldering method were applied. Standard SMD 1206 resistors were used throughout the research, with dimensions as shown in Figure 2.

**Figure 3:**
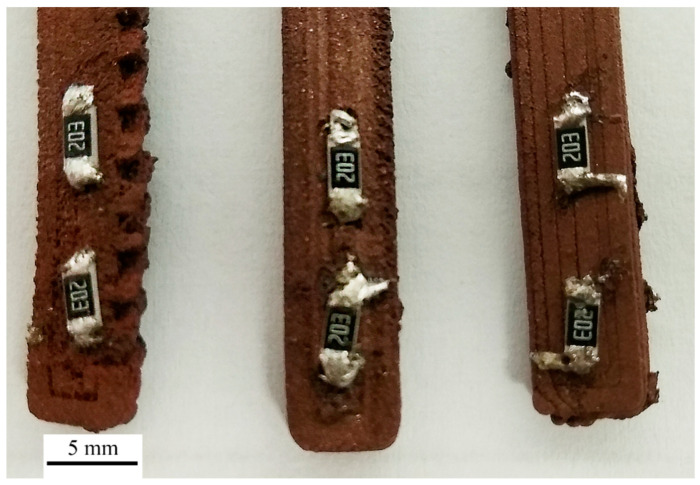
Soldered joints on different composites (from left: ABS/Cu, PS/Cu, PLA/Cu) fabricated with hot iron soldering method and Sn63Pb37 solder alloy.

Hot iron soldering is characterized by the fact that the temperature of the substrate is increased only locally (at the area where the joint is formed), and the process itself can be carried out very quickly, within a few seconds (around 4–6 s), which reduces the impact of high temperature on the composite substrate reducing the risk of thermal damage. On the other hand, SnPb solder alloy makes it necessary to use a soldering temperature value much higher than the softening temperature of polymers used in composites. This is due to the relatively high melting temperature of the SnPb soldering alloy—187 °C. Observations show that it is possible to make such connections thanks to the mechanical pressing of the solder particles into the composite structure. On the other hand, there was no possibility of fabricating repetitive solder joints. The process of hot iron soldering with high temperature causes further damage to each composite (Figure 3). In the case of these connections, it is impossible to determine the contact angle. As a “rule of thumb”, the selection of the solder joints for the further test was based on the preliminary mechanical strength. If resistors were attached to the composite substrate and did not detach during transportation, additional experiments were performed. The soldered joints made on all three types of composite substrates fulfilled this rule. However, it should be noted that the obtained soldered joints have a very irregular and unrepeatable shape. The substrate’s preparation method does not affect the quality of the joints, which is caused by partial damage to the composite substrate during the soldering process.

In the case of the SnPb alloy, it was not possible to perform reflow soldering on composite substrates. In our conductive composites, ABS, PLA, and PS are used as matrix. These polymers have a much lower melting temperature than the melting point of the solder alloy. In contrast to hot iron soldering, where a high temperature is applied selectively at the spot where the soldered joint is made, it is necessary to heat the entire volume of the substrate during reflow soldering in a convection oven in which the elements are soldered. Therefore, composite samples in the convection oven heated to the temperatures above the melting point of the PbSn soldering alloy are destroyed what unable to prepare the soldered joints using this technique.

Another type of soldering alloy used during the research was the low-temperature soldering alloy SnBiAg. In contrast to the previously considered PbSn alloy, the melting temperature of the soldering alloy SnBiAg is lower: 138 °C. The use of a low-temperature soldering alloy makes it possible to significantly reduce the soldering temperature and has the advantage of reducing the risk of damage to composite substrates during both soldering processes.

**Figure 4:**
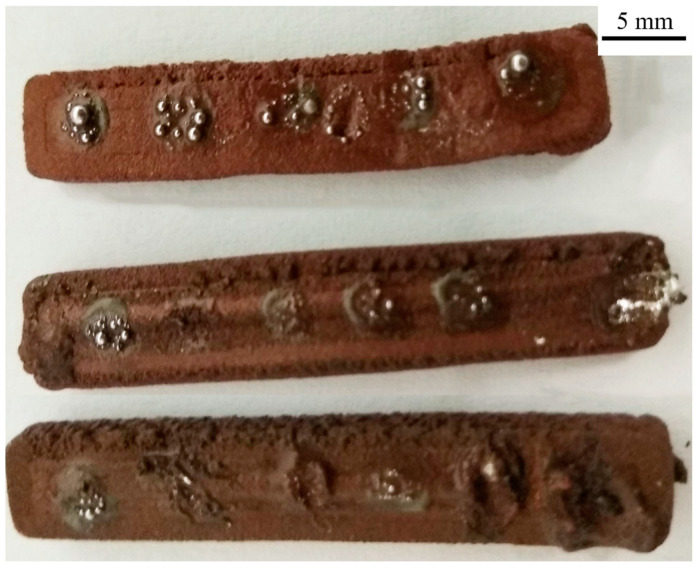
Results of hot iron soldering with low-temperature SnBiAg solder paste on different composites (from top: PLA/Cu, ABS/Cu, Ps/Cu).

Again, attempts have been made to solder SMD resistors to composite substrates using two soldering techniques–hot iron and reflow soldering. Tests show that using the hot iron method with low-temperature SnBiAg soldering paste makes it impossible to obtain satisfying results. The preparation method of the composite surface has no direct impact. The wetting of the composite surface is not good enough, and solder alloy sticks to the hot iron preventing the formation of the soldering joint. The surface energy of fused metal alloys is not compatible with the surface energy of polymer substrate, creating obstacles in the proper wetting of the alloys on printed composited during the soldering process [26]. The greater the substrate surface energy, the better the wettability. The surface energy of polymers is approximately two orders of magnitude lower than metals [27,28,29]. Those material properties result in significantly better wetting of the soldering iron tip than the composite substrate and make it impossible to fabricate solder joints. Soldering attempts were carried out on all three composites without significant positive results (Figure 4).

**Figure 5:**
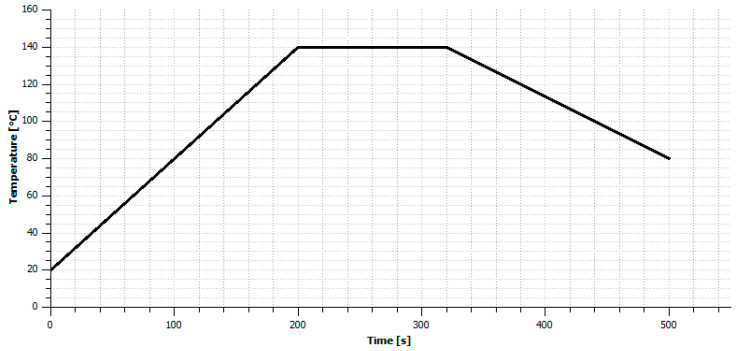
Temperature profile used in reflow soldering process in a convection oven.

Reflow soldering makes it possible to use the temperature closest to the melting point of soldering paste. Thanks to that, the lowest possible temperature necessary to obtain a soldering connection is used in this process. Moreover, additional heating of the substrate on which the solder connection is fabricated helps improve the wetting of the soldering alloy. The soldering process was prepared in the reflow oven, and the reflow temperature profile is shown in Figure 5. Low-temperature SnBiAg paste was applied with the dispenser to ensure the same volume of paste is applied on each sample. Thanks to using low-temperature soldering paste, the maximum temperature used during soldering was set to 140 °C, which is a temperature that does not cause significant damage to the composite substrates used.

Tests of reflow soldering have shown that it is possible to make repeatable joints soldered on all three composite substrates using the SnBiAg low-temperature solder alloy with the reflow soldering technique. All SMD resistors soldered to composite substrates were attached to the substrate, so they were taken into account in further tests of mechanical properties of soldered joints.

**Figure 6:**
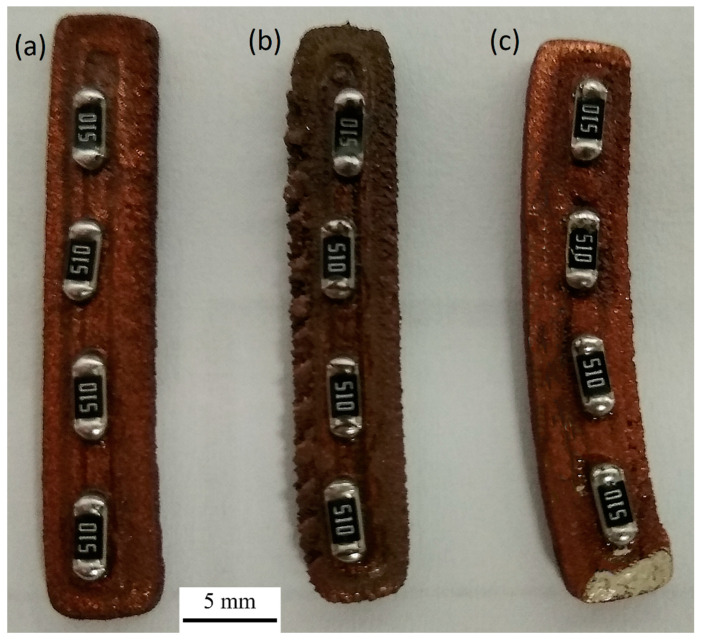
Soldered joint fabricated with reflow soldering on different composites: (**a**) ABS/Cu, (**b**) PLA/Cu, (**c**) PS/Cu.

After reflow soldering tests, we noticed that PS/Cu composite substrates had been deformed in the process (Figure 6). Other composites have not shown such behavior. The deformation of PS/Cu elements can be due to the internal stresses in the material. Thermal stresses can emerge after the printing process and be relieved in the process of slow heating up. Polystyrene has a lower Young’s modulus compared to PLA and ABS. This causes PS/Cu composites to be the easiest to process, especially at higher temperatures. Internal stresses occurring in the material can deform it while relieving. To avoid such deformations during the soldering process on polystyrene matrix composite substrates, selective soldering may be necessary. During the reflow soldering process, the entire volume of the sample was heated to about 140 °C, causing its deformation. The use of another selective soldering method, such as wave soldering or laser soldering, should make it possible to obtain soldered joints without damaging the substrate. Another possible way to avoid such deformations may be to optimize the 3D printing process with PS/Cu composite to prevent an accumulation of thermal stresses on one side of the element. This optimization can be achieved, for example, by randomly choosing the place where the deposition of the layer starts. At this moment, the fabrication of every layer begins in the same X-Y spot. This causes an accumulation of stress on one side of the element. With each layer started in a different spot, deformation should not appear due to the even distribution of stress throughout the element.

While the use of a low-temperature SnBiAg soldering alloy and reflow soldering made it possible to obtain soldered joints with satisfying quality and high repeatability, contact angle measurements were carried out depending on the preparation method of the composite surface. The contact angle measurement results show that the appropriate preparation of the surface is necessary to achieve good quality solder connections. It is noted that the mechanical cleaning of the surface has a significant impact on the contact angle. It is necessary to solder to the copper grains in the composite, not to the polymer matrix, in order to form a correctly soldered joint. The prepared samples are characterized by the fact that during the printing process, they are covered with a layer of polymer that must be removed to expose as much of the copper grains as possible in the place where the soldered joint is formed. After the mechanical removal of polymer film, the solder alloy can be soldered to the copper particles situated below. This is clear for the measured angle values for samples A and D. Moreover, using mechanical cleaning together with additional flux allows fitting the criteria of acceptable wetting θ < 55°, according to Klein-Wassink [30]. The differences in the observed wetting angle values depending on the composite surface preparation are presented in Table 2 on the example of ABS/Cu composite substrates.

This is the first time such experiments measuring the contact angle on conductive composites have been performed. The literature does not provide similar wetting angle results for composite substrates, so it is necessary to refer to standard metal substrates. It must also be noticed that using Pb-free soldering alloys always results in higher contact angle values compared with SnPb soldering alloys [31,32,33]. Another condition that made it challenging to fabricate good-quality solder joints on polymer composite substrate is that higher soldering temperatures positively influence contact angle values [34,35]. In the presented research, the lowest possible soldering temperature was used in order to not damage the composite substrate. Contact angle values from 30° to 58° are reported in the literature for bismuth-based soldering paste, Cu substrate and optimal soldering temperature [32,36,37]. Values in this range were obtained on all tested composite substrates after appropriate substrate preparation (A and D). However, it was observed that the application of mechanical cleaning and suitable flux, with chemical cleaning of the soldered substrate, reduced the average contact angle for reflow soldering significantly. It also resulted in the highest-quality solder joints that fit the criterion of acceptable wetting mentioned earlier. It is worth mentioning that the contact angle value does not vary significantly with different polymer matrix materials in the composite substrate. The most important is the process of surface preparation before the soldering. Table 3 presents the average contact angle values depending on the composite type and surface preparation method. Each average value was calculated from the measurement results of 10 samples.

A brief summary of the quality evaluation of soldered joints depending on the soldering alloy used, the soldering method, and the surface preparation is presented in Table 4. Further tests of the joints’ mechanical properties were carried out only for specimens that passed the “rule of thumb”. Soldering joints that did not pass the “rule of thumb” were classified as not meeting the requirements and were not considered to be soldered joints of acceptable quality.

### 3.2. Shear Test

**Figure 7:**
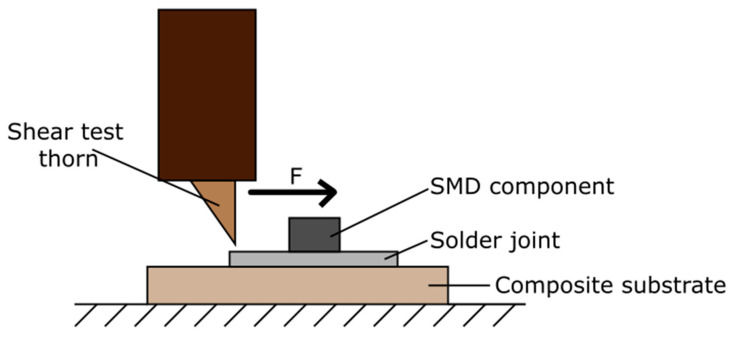
Schematic representation of shear test machine.

**Figure 8:**
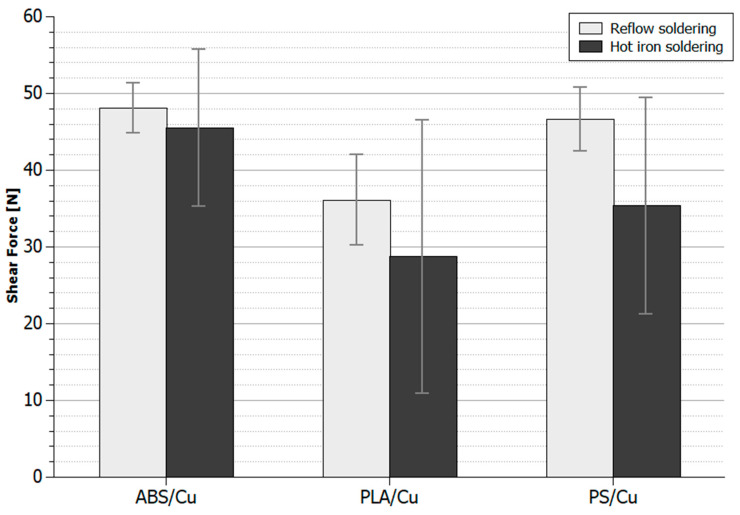
Shear force measurement results of soldering joints. All composite surfaces were mechanically cleaned with sandpaper and RF800 flux was applied before soldering.

According to the wettability test results, two soldering methods were used to fabricate samples for shear tests: hot iron soldering with Sn63Pb37 solder alloy and reflow soldering with OM 520 solder paste. 1206SMD resistors were soldered on each of the composite substrates, ABS/Cu, PLA/Cu, and PS/Cu, using both methods. The schematic diagram of the laboratory stand for investigating the maximum shear force of the solder joints is presented in Figure 7. The thorn applies the shear force on the soldered element until the component, joint, or substrate is damaged. The maximum value of the shear force used during the test was measured. For all tested components, the connection was damaged between the surface of the composite and the solder. There were no cases in which the SMD component or composite paths were damaged. The measurement results are presented in Figure 8.

It was also noticed that soldering joints prepared with the hot iron method significantly differed from each other, with a noticeable spread of measured shear force values. These results confirm the previous observation that the quality of soldering joints fabricated with this method is variable. The significant differences in the shear force measurement results obtained are also related to the impossibility of fabricating surface-repeatable connections soldered with a soldering iron. It should also be mentioned that a solder joint is only formed with the surface of the copper, not the polymer matrix, in the composite. The spread of the obtained results is also due to the variable number of copper grains on the composite surface where the soldering process was performed.

The results of shear force measurements also correspond with the thermal stability properties of the polymer matrix. It can be observed that for composites based on polymer matrixes with lower glass transition temperatures, the maximum value of the force at solder connection failure is lower. Materials with lower glass transition temperatures show lower thermal stability, and thermal degradation is quicker, resulting in failure at lower shear forces (Table 5).

Comparing the results obtained with other examples of soldering tests on 3D-printed composite substrates is not possible, because there are no such reports in the literature. Therefore, the obtained results were compared with the shear force values for standard joints soldered on metal substrates. The shear force reported in the literature, depending on the soldering paste used, for the 1206 SMD composite size soldered to a copper surface varies from 86 to 111 N [38,39]. The shear forces obtained for polymer composites as substrates are around 50% lower. The mechanical properties of the prepared soldered joints are still good enough to prepare high-quality electrical connections that easily pass the “rule of thumb”. Developing a method for soldering standard electrical components to 3D-printed composites allows us to merge traditional electronics with the rapidly growing field of structural electronics. It is also worth mentioning that structural electronics are expected to embed electronic elements in the housing of structural materials with excellent mechanical properties [40]. Therefore, the mechanical properties of electrical connections are of secondary importance, because components in structural electronics systems will be sealed inside the structure of the whole device and thus protected from forces that could damage the electrical connections between the components.

### 3.3. Demonstrator Fabrication

**Figure 9:**
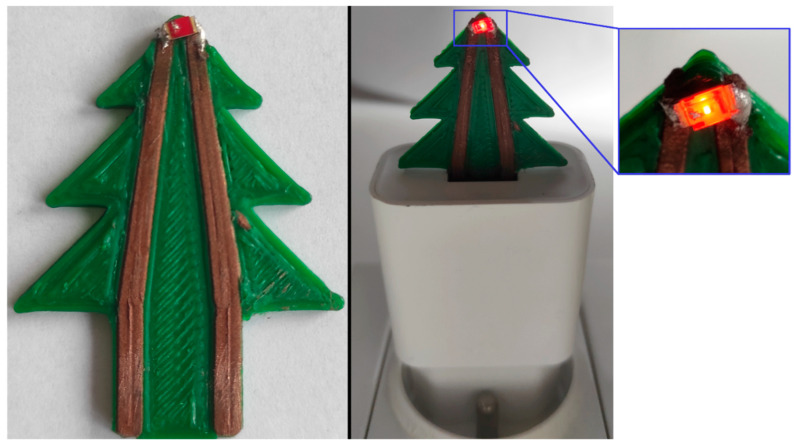
3D-printed demonstrator with soldered LED on PS/Cu conductive substrate using a hot iron and SnPb soldering alloy.

To demonstrate the functionality of the developed conductive composites together with the soldering method, the electrical structure was fabricated. Conductive paths were 3D printed on an unmodified FDM printer, and an SMD LED component was soldered to its surface (Figure 9). Conductive paths were printed with PS/Cu composite filled with 85.5 wt.% copper powder. The LED was soldered to the surface using the hot iron method with Sn63Pb37 solder alloy. Previous tests showed that reflow soldering gave far better results, but using the hot iron method in demonstrator fabrication proved that it could also be used in the fast prototyping of structural electronics systems. A 5 V USB charger powered the electronic circuit. The electrical resistivity of the printed composites and solder connections was low enough to power the LED with high efficiency. The example of this demonstrator shows that it is possible to manufacture complex electronic systems using additive techniques despite their apparent limitations. The limitation of obtaining miniature integrated circuits with FDM technology can be solved by combining printed elements with standard electronic components. Soldering the components to composite substrates turns out to be a method that can be used to obtain fully functional 3D-printed electronic systems.

## 4. Conclusions

The possibility of fabricating soldering joints on 3D-printed conductive substrates was investigated in the current work. Two soldering methods (hot iron soldering and reflow soldering) and two soldering alloys (SnPb and low-temperature SnBiAg) were tested to find the optimal technique for soldering. The main conclusions can be summarized as follows:

1. The surface preparation of conductive composite plays a significant role in forming high-quality solder joints. The influence of surface preparation was evaluated by measuring the contact angle of the melted solder alloy. It can be observed that the best results were obtained with the mechanical removing of a thin polymer layer, revealing a copper powder inside a composite structure, and using flux that allows the removal of oxides and other compounds from the soldered surfaces. After the surface preparation, the contact angle value was reduced to 55° for reflow soldering with low-temperature SnBiAg solder paste, which fits the criteria of acceptable wetting.
2. The shear test results show that the maximum force value that could be applied before damaging soldered joints is strongly dependent on the polymer matrix used in the composite substrate. In general, maximum shear force values for reflow soldering are slightly higher than for hot iron soldering. Shear forces obtained for polymer composites as substrate are around 50% lower compared to a typical copper substrate.
3. The highest average shear strength of the joint occurs on ABS/Cu substrates, then on PS/Cu, and the lowest results were obtained for the PLA/Cu substrate. The obtained results correlate directly with the thermal stability of the polymer matrixes used. The higher the thermal stability of the substrate material, the higher the maximum shear force of the soldered joints fabricated on that substrate.
4. Hot iron soldering can be used to fabricate soldered joints on prepared composite composites. Still, it is recommended that this method only be used for rapid prototyping of individual structures because of its low repeatability.
5. The soldering joints with the best properties were obtained using low-temperature SnBiAg solder paste and reflow soldering. This allows the fabrication of repeatable, high-quality solder joints without damaging the composite substrate. This method can be used for the mass production of structural electronics elements.

## Figures and Tables

**Table 1:** List of preparation methods of the composite surfaces before soldering.

Surface Section Symbol	A	B	C	D	E
**A method of surface preparing**	Surface mechanically cleaned with 400 grit sandpaper	RF800 flux applied on surface before soldering	Surface chemically cleaned with solvent	Surface mechanically cleaned with sandpaper and RF800 flux applied before soldering	No surface preparation before soldering

**Table 2:** Contact angle measurements results of SnBiAg solder correlated with the surface preparation of ABS/Cu composite.

Surface Section Symbol	A	B	C	D	E
**A Method of Surface Preparing**	Surface mechanically cleaned with 400 grit sandpaper	RF800 flux applied on surface before soldering	Surface chemically cleaned with solvent	Surface mechanically cleaned with sandpaper and RF800 flux applied before soldering	No surface preparation before soldering
**Solder Ball**	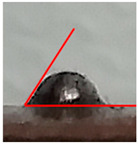	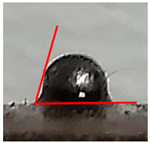	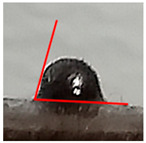	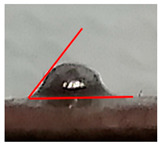	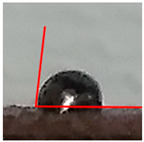
**Edge** **Detection**	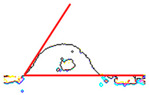	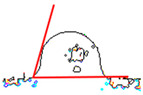	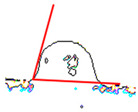	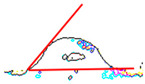	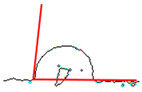
**Measured Contact** **Angle**	57°	74°	78°	50°	83°

**Table 3:** Average contact angle measured values for SnBiAg soldering alloy and reflow soldering technique.

Matrix Polymer	Surface Preparation Method				
	A	B	C	D	E
**ABS**	57.82° ± 0.85°	74.48° ± 0.82°	78.07° ± 0.81°	50.27° ± 0.95°	83.74° ± 1.15°
**PLA**	57.24° ± 0.85°	74.09° ± 0.98°	78.18° ± 0.79°	50.48° ± 0.91°	84.02° ± 1.19°
**PS**	57.57° ± 1.11°	74.34° ± 0.76°	78.48° ± 0.77°	50.55° ± 0.81°	84.11° ± 1.18°

**Table 4:** Impact of the soldering alloy and soldering method on the quality of the soldered joints.

Solder Alloy	Soldering Method	Description	Fit the Criteria of Acceptable Wetting (θ < 55°)
SnPb	Hot iron soldering	Possible to fabricate soldered joints on all tested composite substrates; the method of surface preparation does not affect the quality of joints; not possible to determine the wetting angle; irregular shape of the obtained soldered joints	yes
SnPb	Reflow soldering	Not possible to fabricate soldered connections due to the high melting point of the soldering alloy (significantly exceeding the softening temperature of the polymer matrixes in all tested composites)	no
SnBiAg	Hot iron soldering	Not possible to fabricate solder joints due to the significant difference in wettability between the composite substrates and the soldering tip; soldering alloy sticks to the soldering tip and does not deposit on the composite substrate	no
SnBiAg	Reflow soldering	Possible to fabricate repeatable soldered joints; quality of soldered joints depends mainly on how the composite substrate’s surface is prepared; minor impact of the type of composite matrix on the quality of joints	yes

**Table 5:** Glass transition temperature for different polymers according to literature.

Polymer	Glass Transition Temperature (T_g_) [°C]	Average Shear Force (Reflow Soldering) [N]	Average Shear Force (Hot Iron Soldering) [N]
ABS	105	48.1	45.5
PS	100	46.7	35.4
PLA	60	36.1	28.7

## Data Availability

Data is contained within the article.
